# Psychometric Properties of the Translated Tai Chi Exercise Self-Efficacy Scale for Chinese Adults with Coronary Heart Disease or Risk Factors

**DOI:** 10.3390/ijerph18073651

**Published:** 2021-03-31

**Authors:** Ting Liu, Aileen Wai Kiu Chan, Ruth E. Taylor-Piliae, Kai-Chow Choi, Sek-Ying Chair

**Affiliations:** 1 The Nethersole School of Nursing, Faculty of Medicine, The Chinese University of Hong Kong, Shatin, New Territories, Hong Kong; liuting@link.cuhk.edu.hk (T.L.); kchoi@cuhk.edu.hk (K.-C.C.); sychair@cuhk.edu.hk (S.-Y.C.); 2 College of Nursing, The University of Arizona, 1305 N. Martin, P.O. Box 210203, Tucson, AZ 85721-0203, USA; rpiliae@arizona.edu

**Keywords:** Tai Chi, exercise self-efficacy, exercise behavior, physical activity, coronary heart disease, cross-cultural adaptation, translation, reliability and validity

## Abstract

Tai Chi is an effective exercise option for individuals with coronary heart disease or its associated risk factors. An accurate and systematic assessment of a Mandarin-speaking adults’ self-efficacy in maintaining Tai Chi exercise is lacking. Mandarin Chinese has the most speakers worldwide. This study aimed to translate the Tai Chi Exercise Self-Efficacy scale and examine its psychometric properties. The 14-item Tai Chi Exercise Self-Efficacy scale was translated from English into Mandarin Chinese using a forward-translation, back-translation, committee approach, and pre-test procedure. Participants with coronary heart disease or risk factors (*n* = 140) enrolled in a cross-sectional study for scale validation. Confirmatory factor analysis indicated a good fit of the two-factor structure (Tai Chi exercise self-efficacy barriers and performance) to this sample. The translated scale demonstrated high internal consistency, with a Cronbach’s α value of 0.97, and good test-retest reliability, with an intra-class correlation coefficient of 0.86 (*p* < 0.01). Participants with prior Tai Chi experience reported significantly higher scores than those without (*p* < 0.001), supporting known-group validity. A significant correlation was observed between the translated scale and total exercise per week (r = 0.37, *p* < 0.01), providing evidence of concurrent validity. The Mandarin Chinese version of the Tai Chi Exercise Self-Efficacy scale is a valid and reliable scale for Chinese adults with coronary heart disease or risk factors.

## 1. Introduction

Coronary heart disease (CHD) persists as the leading cause of mortality and disability globally [1,2]. In China, CHD-related mortality has increased rapidly during the past decade. In 2016, the mortality rates were 113.46 and 118.74 per 100,000 population in urban and rural areas, respectively. These rates were much higher than those in 2002, at 39.56 and 27.57 per 100,000 population, respectively [3]. In China, CHD affected an estimated of 11 million people in 2018, and its prevalence rate continuously increases [3]. Various cardiovascular risk factors have contributed to the increased likelihood of developing CHD, such as age, family history of CHD, diabetes, hypertension, hyperlipidaemia, being a current smoker, unhealthy diet, obesity, or physical inactivity [4,5]. The more risk factors an individual has, the greater chance they have for developing CHD.

Physical inactivity is a modifiable risk factor for CHD, and it may lead to hypertension, diabetes, dyslipidaemia, being overweight, or obesity [5]. Regular participation in adequate levels of physical activity is associated with a decreased risk of developing CHD, along with improvements in cardiorespiratory fitness and psychological stress [6]. Regular exercise, such as aerobic exercises or traditional Chinese exercises, for instance Tai Chi, contributes to a reduction in cardiac mortality [7,8,9]. The American Heart Association (AHA) recommend that adults complete at least 150 min/week of moderate exercise or 75 min/week of vigorous exercise, or a combination of both, to improve overall cardiovascular health [10]. However, a recent national survey in China reported that only 22.8% adults (aged from 20–59) met this physical activity recommendation in 2014, reflecting the difficulties and complexities for personal behavioral change [11].

Tai Chi, a popular exercise worldwide and especially among the Chinese population, is a mind-body exercise that combines harmonious body movements with controlled breathing [12]. In view of the potential physical and psychological benefits of Tai Chi, Tai Chi is a promising exercise option for CHD management. Based on a systematic review and meta-analyses, Tai Chi groups showed significant improvements in cardiorespiratory fitness and psychosocial well-being, with improvements in anxiety, depression, and health-related quality of life in patients with CHD when compared with no-treatment control groups [13]. Among the various designs of the Tai Chi interventions, at least 2–3 Tai Chi exercise sessions per week (60 min per session) for 12 weeks led to significant improvements in these outcomes [13]. In addition, a 12-week Tai Chi intervention with 2-3 Tai Chi sessions per week (60 min/session) can also be beneficial in reducing cardiovascular risk factors, such as hypertension, elevated fasting blood glucose, and being overweight, among adults with cardiovascular diseases or cardiovascular risk factors [14,15,16]. Tai Chi is an exercise that does not require any equipment and can be practiced at home according to an individual’s schedule so that common barriers, such as lack of accessibility, transport problems, and work or schedule conflicts to exercise, can all be eliminated [17,18]. Therefore, Tai Chi is a relevant and beneficial intervention strategy for adults with CHD or risk factors.

Exercise is closely associated with an individual’s thoughts and beliefs about whether they have the ability to perform the activities that influence their behavior. These beliefs were labelled by Bandura as self-efficacy, representing an individual’s confidence in using their skills and abilities to perform a certain behavior [19]. Thus, the more self-efficacy an individual has, the more confidence they have in accomplishing the specified behavior. Given that perceived self-efficacy is concerned with one’s belief in one’s abilities to achieve a specified behavior, self-efficacy scales must be tailored to a specific behavior [20].

Tai Chi exercise self-efficacy (TCSE) refers to beliefs in an individual’s ability to overcome barriers and perform Tai Chi. Thus, a higher score on the TCSE scale indicates higher confidence or perceived self-efficacy. The original English version of TCSE scale was previously translated and used among Cantonese-speaking adults living in the United States, with a high reported internal consistency (TCSE performance, α = 0.97; TCSE barriers, α = 0.95) [21]. Known-groups validity was also established with significant differences between the participants who reported prior Tai Chi experience and those who reported no prior Tai Chi experience [21].

To promote exercise behavior and physical activity, it is useful to consider self-efficacy-related interventions to boost an individual’s efficacy beliefs, given that these beliefs are the major determinants of an individual’s choice of activities, how much effort he/she will expend, and how long he/she will sustain the effort in dealing with stressful situations [22]. The practice of Tai Chi to reduce the burden of chronic diseases and improve quality of life is growing in popularity among the elderly in mainland China. Tai Chi interventions could be structured by building upon the TCSE scale to enhance self-efficacy beliefs among the elderly to maintain regular exercise behavior and then achieve good health-related outcomes. Moreover, Mandarin Chinese has the most speakers in the world of any language [23]. Yet, an accurate and systematic assessment of TCSE for use in mainland China is lacking. Moreover, the Cantonese-language version of the TCSE scale, which was validated among ethnic Chinese with CHD risk factors in the United States, is not appropriate for Mandarin-speaking adults. Although Cantonese and Mandarin are etymologically related and grammatically similar, their written languages use different characters. In certain cases, they use different lexicons to express the same meaning, and different cultural factors that need consideration exist [24,25]. The cross-cultural adaptation of a self-administered scale for use in a new language and culture requires a unique methodology to obtain content and semantic equivalence between the original and target languages. Hence, this study aimed to translate the original English version of the TCSE scale into Mandarin Chinese and to validate the Mandarin Chinese version of TCSE for use among adults with CHD or CHD risk factors living in mainland China.

## 2. Methods

### 2.1. Participants and Settings

This cross-sectional study was conducted from June to September 2017. The study sample was recruited from one community health center in Jinan, mainland China. The inclusion criteria were people who had CHD or at least three of the following CHD risk factors [4,12]: (1) older than 55 years older (female), or postmenopausal, or older than 45 years older (male), (2) hypertension or taking blood pressure medication, (3) diabetic or taking medicine to control blood sugar, (4) hyperlipidaemia, (5) current Smoker, (6) physically inactive (≤30 min of moderate-intensity physical activity for ≥3 days per week for ≥3 months [26]), (7) waist circumference ≥ 80 cm (female) or ≥90 cm (male) or body mass index >25 kg/m^2^. Participants were excluded if they had a severe sensory or cognitive impairment (a score on the Abbreviated Mental Test of more than 7) [27], or unable to answer the questions and provide informed consent.

The sample size of the current study was determined to provide an adequate number of participants for conducting a factor analysis of the TCSE. According to the widely accepted rule of thumb for the sample size requirements for factor analysis [28], at least 10 participants per item of a scale are needed. Hence, a sample size of 140 study participants was recruited to evaluate the psychometric properties of the 14-item TCSE. Furthermore, a sample size of 140 participants would provide sufficient power to detect a correlation coefficient with values of as low as 0.23 at 80% power and a 5% level of significance for assessing concurrent validity of the TCSE.

Based on the methods and sample size formula of Bonett [29], a sub-sample of 50 participants was determined to be adequate for examining test-retest reliability by using intraclass correlation coefficients (ICC) at a 5% level of significance with an anticipated reliability of ICC ≥ 0.8.

### 2.2. Study Instruments

The original 14-item English version of TCSE is a self-administered scale and consists of two subscales (Table 1), with the first subscale (items 1 to 9) being used to assess perceived self-efficacy to overcome barriers to practicing Tai Chi (TCSE barriers) and the second subscale (items 10 to 14) being used to evaluate self-efficacy in performing Tai Chi (TCSE performance) [21]. The response options of each item range from 0 to 100 (0 = not at all confident, 100 = very confident). The combined score for each sub-scale is the sum score of all items in each sub-scale, which is then divided by the total number of items in each sub-scale. A higher score represents higher perceived self-efficacy.

**Figure 1:**
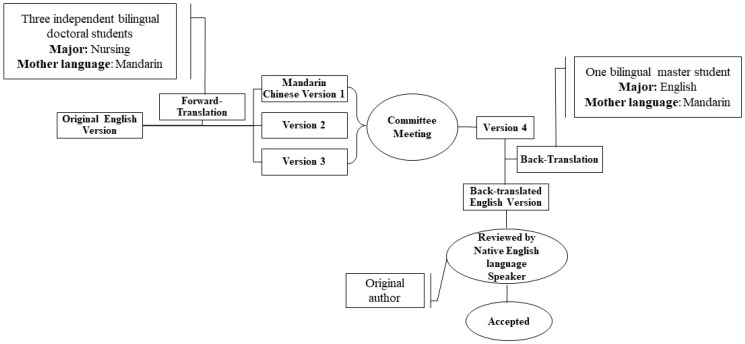
Translation Process of the Tai Chi Exercise Self-Efficacy scale.

With permission from the developer of the TCSE scale, the Chinese version for Mandarin speakers in mainland China was translated from the original 14-item English version of TCSE in accordance with previous translation guideline [28] and Cha’s combined translation techniques [30]. The translation procedure included forward-translation and back-translation methods, a committee approach, and pre-testing by using a monolingual sample to establish semantic equivalence (Figure 1).

The first step in the process was forward translation. This step included three independent bilingual nursing doctoral students whose mother language was Mandarin. They were asked to independently translate the English version into a Mandarin Chinese version. A committee meeting was then conducted to discuss differences between the translated versions. This procedure was continued until all three translators agreed on the final translated instrument. The revised translated Mandarin Chinese version was then back-translated into English. An independent bilingual master-level student from mainland China who was proficient in English was invited to independently complete the back-translation process. Some minor differences were found between the original English version and the version that was back-translated from Mandarin to English. For example, item 3 was “I feel pain or discomfort when doing Tai Chi” in the original English version, was back-translated from Mandarin to English as “My body feels painful or comfortless when doing Tai Chi”. The back-translated version was sent to a Native English language speaker, that is, the original TCSE developer, for comparison of the original English version with the back-translated English version to determine whether these minor differences would be accepted. The back-translated version of the TCSE scale was found to be acceptable without requiring any further changes. Hence, the translated Mandarin Chinese version of TCSE was found to have obtained initial conceptual, semantic, and content equivalence [28].

Next in the translation process, a total of 10 participants were invited to rate the instructions, response formats, and items of the scale using a dichotomous scale (i.e., clear or unclear) [28]. As determined a priori, the instructions, response format, and items of the scale that were found to be unclear by ≥20% of the participants would be re-assessed. All of these participants (*n* = 10) rated the instructions and response format as clear, and 90% (*n* = 9) rated the scale items as clear. This step provided further support for the semantic and content equivalence of this translated scale.

In addition, a panel of five experts that included one cardiologist and four geriatric nurse specialists were invited to evaluate each item of the TCSE for content relevance by using a four-point Likert scale, from 1 (not relevant) to 4 (highly relevant). The content validity index (CVI) at the item level (I-CVI) and scale level (S-CVI) were calculated to examine the content validity of the translated scale. An I-CVI of 0.8 or above and an S-CVI of 0.9 or above were regarded to be acceptable for content validity [28]. All five expert panel members submitted their ratings, without any missing responses. The I-CVIs ranged from 0.80 to 1, and the S-CVI was 0.96. The results indicated that the Mandarin Chinese version of TCSE had acceptable content validity. The expert panel also indicated that this scale was conceptually and culturally relevant to measure TCSE in Mandarin Chinese speakers with CHD or CHD risk factors.

A demographic questionnaire was administrated to collect the participants’ demographic characteristics, such as gender, age, smoking history, medical history, exercise frequency, and prior Tai Chi experience.

### 2.3. Data Collection

Ethical approval was obtained from the Survey and Behavioral Research Ethics Committee of the university. The study was conducted in accordance with the principles outlined in the Declaration of Helsinki. Informed consent was obtained from each eligible participant after the researcher had provided a thorough explanation of the study. Additionally, the TCSE was administered two weeks later to a random sub-sample of 50 participants, who were part of the 140 initial participants, to examine the test-retest reliability of the translated TCSE scale.

### 2.4. Data Analysis

Version 25 of SPSS was employed to perform the data analyses. The appropriate descriptive statistics were used to summarize the demographic and clinical characteristics of the participants. Frequency and percentage were applied to summarize categorical variables, and means and standard deviation were used for continuous variables. The statistical values of skewness and kurtosis were employed to examine the normality distribution of continue variables. Values for skewness and kurtosis within the range of −2 to +2 were considered to be acceptable [31]. All statistical tests involved were two-sided, with the significant level α set at 0.05.

A confirmatory factor analysis (CFA) was conducted using LISREL 8.8 (Scientific Software International, Inc., Chicago, IL, USA) to examine the factorial validity of the TCSE (Mandarin Chinese version). The following fit indices were used to assess the goodness-of-fit of the model: the chi-square statistic to degree of freedom ratio (χ^2^/df), the root mean square error of approximation (RMSEA), standardized root mean square residual adjusted (SRMR), the comparative fit index (CFI), the goodness-of-fit index (GFI), and the adjusted goodness-of-fit index (AGFI). Acceptable model fit was indicated by a χ^2^/df ratio <3, SRMR value of ≤0.1, RMSEA value of ≤0.08, CFI ≥ 0.90, GFI ≥ 0.90, and AGFI ≥ 0.85.

The Cronbach’s α was computed to evaluate the internal consistency of the translated scale. A Cronbach’s α coefficient of 0.7 or above is considered acceptable, and values ≥ 0.8 are preferable [32,33]. The corrected item-total correlation was applied to identify any items that were non-homogenous with other items in testing TCSE [34]. Items that had a corrected item-total correlation below 0.4 and whose deletion led to a raising of 0.1 or above in the α coefficient for the whole scale were considered as non-homogenous and were dropped [35].

Test-retest reliability refers to the ability of the scale to provide the same results in separate situations and was assessed by using the ICC [36]. The ICC can vary between 0 and 1.0, and an ICC between 0.4 and 0.75 indicates general to good reliability, while above 0.75 shows excellent test-retest reliability [37].

The construct validity concerns the ability of an instrument to measure the construct it is intended to measure [36] and was examined by known-group comparisons in this study. Known-group comparisons were evaluated by using an independent *t*-test for the total score of the TCSE and the score of the TCSE barriers, which exhibited normal distribution. The Mann–Whitney U test was used for the score of TCSE performance. The TCSE of participants with prior Tai Chi experience were compared with those of participants without prior Tai Chi experience on the basis of the premise that having a direct experience of Tai Chi mastery is of foremost importance to higher self-efficacy [20,38]. Construct validity was supported if study results were consistent with the assumption.

Concurrent validity was evaluated by testing the correlations between TCSE and exercise behavior [39]. A positive correlation between the TCSE score and the total exercise time per week was expected as a priori. Total exercise time was assessed by using two questions regarding weekly exercise frequency (days) and average exercise duration (hours) each day. The total exercise time per week was calculated by multiplying the responses from these two questions.

## 3. Results

### 3.1. Sample Characteristics

In total, 140 community-dwelling adults with CHD or CHD risk factors were recruited. The demographic and clinical characteristics of the participants are summarized in Table 2. The average age of the sample was 64.5 (SD = 8.1) years old, and 53 (37.9%) were males. One-third (*n* = 46, 32.9%) reported being diagnosed with CHD, and more than one-third (*n* = 52, 37.1%) reported having hypertension, which was the most common risk factor among the participants. A total of 51 (36.4%) participants reported prior Tai Chi experience. The time for completing the TCSE (Mandarin Chinese version) ranged from two to five minutes. The average total score of the TCSE was 62.1 (SD = 31.1). The TCSE barriers mean score was lower (mean = 48.7, SD = 40.0) than the TCSE performance mean score (mean = 86.3, SD = 25.9) (Table 2).

### 3.2. Factorial Validity

**Figure 2:**
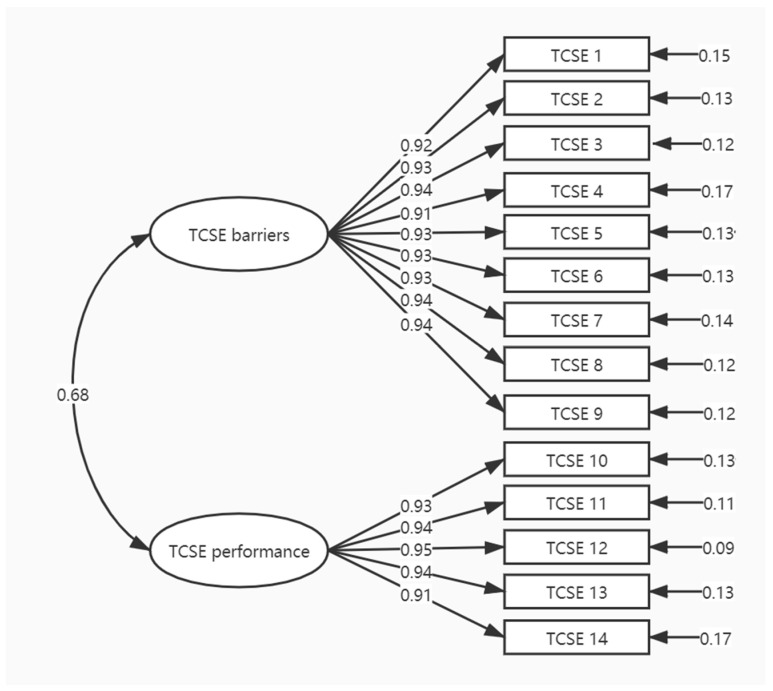
Confirmatory factor analysis model for the TCSE scale (Mandarin Chinese version).

CFA was conducted to examine the two-factor structure (TCSE barriers and TCSE performance) (Figure 2) as suggested by the original version of the scale. The CFA results indicated that this factor structure is a fairly good fit to our data (the χ^2^ of the model was 82.55 with df = 76, *p* = 0.28; the goodness-of-fit indices RMSEA = 0.025, CFI = 1.00, GFI = 0.92, AGFI = 0.89, and SRMR = 0.02).

### 3.3. Reliability

The Cronbach’s α of the translated TCSE was 0.97, which was high and indicated a robust internal consistency. For the two factors (sub-scales), the TCSE barriers and TCSE performance were 0.99. Table 3 shows the item-to-total correlations. All items in the TCSE were above the criterion level of 0.40 [35], which indicated the items’ homogeneity in measuring the concept of TCSE. Additionally, the Cronbach’s α of the translated scale did not increase by more than 0.10 upon the deletion of any item. A sub-sample of 50 participants (participants with prior Tai Chi experience = 26; without prior Tai Chi experience = 24) were reassessed to examine the test-retest reliability of the translated TCSE. The ICC for the scores of the TCSE barriers, TCSE performance, and total TCSE scale were 0.86, 0.68, and 0.86 (all *p <* 0.001), respectively, showing satisfactory test-retest reliability.

### 3.4. Construct Validity

As hypothesized, participants with prior Tai Chi experience (*n* = 51) showed significantly higher TCSE mean scores than participants without prior Tai Chi experience (*n* = 89) (all *p*-values < 0.001, Table 4), providing evidence of known-groups validity.

### 3.5. Concurrent Validity

The scores of the TCSE barriers, TCSE performance, and the total TCSE scale had statistically significant positive correlations with the total exercise score (r = 0.37, 0.47, and 0.42 respectively, *p <* 0.01). These findings indicate initial concurrent validity of the translated TCSE.

## 4. Discussion

This was the first study to translate the original English language version of the TCSE scale into Mandarin Chinese and examine its psychometric properties for use among mainland Chinese adults with CHD or CHD risk factors. Tai Chi has become increasingly popular amongst all age groups and is estimated to have the largest number of exercise practitioners in the world [14]. Given that cardiovascular healthcare providers play an important role in promoting physical activity, managing cardiovascular risk factors, and promoting a healthy lifestyle among CHD patients, measuring the TCSE of individuals with CHD or CHD risk factors is important for clinical practice and research for several reasons. First, by building upon this scale, healthcare providers could structure Tai Chi interventions to optimize beliefs among patients with CHD or CHD risk factors to maintain regular physical activity by incorporating skills to build self-efficacy. Secondly, the TCSE scale provides healthcare providers with a standardized, valid, and reliable tool for evaluating Tai Chi interventions. The results of this study indicated that this translated Mandarin Chinese version of TCSE was linguistically and culturally acceptable for use among the Mandarin-speaking population. If scales developed in one culture (e.g., the United States) are to be used across cultures, then the items in these scales must be accurately translated to capture the linguistic and cultural aspects of the target population to conduct cross-cultural research [28].

For the psychometric properties of the translated TCSE, the results of the CFA supported the consistency of the two-factor structure of the scale (Mandarin Chinese version, comprising TCSE barriers and TCSE performance) with the original English language scale [21]. In line with Bandura’s concept of self-efficacy, the measure of exercise self-efficacy requires respondents to evaluate their confidence in their capability to exercise in the context of potential barriers [40]. Hence, the TCSE barriers subscale (items 1 to 9) refers to participants’ perceived confidence in practicing Tai Chi exercise three times/week when faced with various situations, such as feeling pain or discomfort when doing Tai Chi or being too busy with other activities, that are known to impede exercise participation [41]. On the other hand, task self-efficacy involves one’s belief that one can accomplish a single instance of a defined behavior at different levels of performance [40]. Therefore, the TCSE performance subscale (items 10 to 14) relates to one’s perceived ability to move one’s body in a slow, rhythmical, and continuous fashion for 2 to 30 min [42,43]. Both of the TCSE subscales represent an individual’s confidence in using their skills and abilities to overcome barriers and perform Tai Chi exercise.

The results indicate that the internal consistency of the translated Mandarin Chinese version of the TCSE was high for both subscales (TCSE barriers, Cronbach’s α = 0.99; TCSE performance, Cronbach’s α = 0.99), and was slightly higher than that of the Cantonese language version of the TCSE (TCSE barriers, Cronbach’s α = 0.95; TCSE performance, Cronbach’s α = 0.97) used among ethnic Chinese with CHD risk factors living in San Francisco [21,44]. A very high Cronbach’s alpha may indicate that some items in a scale may be redundant [21]. Nevertheless, a number of factors can influence Cronbach’s alpha, such as selectivity of the sample and number of subjects [41,45]. The current study sample was 140, and all were recruited from one community health center. Thus, future studies may consider recruiting a larger sample from diverse settings to further examine the TCSE scale’s internal consistency. Furthermore, the test-retest scores in this study revealed that the translated Mandarin Chinese version of TCSE was stable and produced consistent results.

Consistent with a prior study [21], the current work identified significant differences between the TCSE scores of participants with prior Tai Chi experiences and those without, and provided evidence for known-groups validity. Bandura identified the four sources of efficacy beliefs, namely mastery experiences, vicarious experiences, verbal persuasion, and emotional and physiological states [20,38]. Among these four sources, having a direct experience of mastery experiences is regarded as the most important source for building and fostering one’s confidence in performing a specific behavior. Previous Tai Chi experiences may have helped build or foster the practitioners’ confidence in overcoming barriers and performing Tai Chi, and this explains, in part, the current results. Tong et al. supported the notion that performing Tai Chi leads to mastery experience, which increases one’s confidence in their abilities [46].

Consistent with previous validation studies [21,39,47], significant associations were observed between TCSE (Mandarin Chinese version) scores and total exercise per week. The direction of this correlation was hypothesized a priori, providing evidence for the concurrent validity of the translated TCSE.

This study, nevertheless, had some limitations. First, all study participants were recruited from only one community health center in mainland China. This approach limits the generalizability of the study results. Second, the method used to calculate the total exercise per week in our study (i.e., multiplying the weekly exercise frequency (days) and average exercise duration (hours) each day) excluded the intensity of the exercise performed. While we found initial evidence for the validity and reliability of the TCSE scale (Mandarin Chinese version), it has only been examined among adults with CHD or CHD risk factors. Further research using the Mandarin Chinese version of TCSE and examining its psychometric properties among healthy adults or those with other chronic diseases is recommended.

## 5. Conclusions

Exercise self-efficacy is a significant cognitive mediator in the maintenance of exercise behavior. The measure of exercise self-efficacy needs to be tailored to a specific behavior (e.g., Tai Chi). The current study provided initial evidence that the Mandarin Chinese version of TCSE is a reliable and valid measure that is appropriate for use among Mandarin-speaking adults with CHD or CHD risk factors. Our study confirmed a two-factor structure of the TCSE, which can be used to assess TCSE barriers and TCSE performance. In summary, the TCSE is brief, easy to understand and administer, and user-friendly. It is important for understanding and assessing the confidence of adults with CHD or CHD risk factors for engaging in Tai Chi exercise in mainland China. Having a valid and reliable measure assessing TCSE could help frame Tai Chi interventions to motivate individual to initiate and adhere to regular exercise programs.

## Figures and Tables

**Table 1:** Items of TCSE scale (original English version and Mandarin Chinese version).

English	Mandarin Chinese Version
**I believe I can perform Tai Chi exercise three times per week, even if……**	**我相信即使在下列情况发生时, 我也可以每周练习太极三次…**
1. The weather was bothering me?	天气使我感到困扰时?
2. I am bored by Tai Chi?	我对太极感到无趣时?
3. I feel pain or discomfort when doing Tai Chi?	当我在练习太极时, 我感到疼痛或者不舒服?
4. I have to perform Tai Chi alone?	我得独自练习太极时?
5. I do not enjoy Tai Chi?	我不享受练习太极时?
6. I am too busy with other activities?	我太忙于其他活动时?
7. I feel tried?	我感到疲惫时?
8. I feel stressed?	我感到紧张或者有压力时?
9. I feel depressed?	我感到沮丧或情绪低落时?
**I believe I can move my whole body in a slow, rhythmical fashion continuously…**	**我相信我可以用一种缓慢, 有节奏的方式持续移动我的整个身体…**
10. For 2 min	长达2分钟
11. For 5 min	长达5分钟
12. For 10 min	长达10分钟
13. For 20 min	长达20分钟
14. For 30 min	长达30分钟

Note: TCSE = Tai Chi exercise self-efficacy. Original English scale derived from Taylor-Piliae, R.E. and Froelicher, E.S. Measurement properties of Tai Chi exercise self-efficacy among ethnic Chinese with coronary heart disease risk factors: a pilot study. *Eur. J. Cardiovasc. Nurs.* **2004**, *3*, 287–294. Used with permission.

**Table 2:** Characteristics of the Chinese participants with CHD or CHD risk factors (*n* = 140).

Characteristics	Mean ± SD or *n* (%)
Age (years)	64.5 ± 8.1
Gender	
Male	53 (37.9%)
Female	87 (62.1%)
BMI (kg/m^2^)	25.3 ± 2.9
Education level	
No formal education	7 (5.0%)
≤Primary	68 (48.6%)
≤Secondary	49 (35.0%)
Tertiary	16 (11.4%)
Marital status	
Married	132 (94.3%)
Divorced	8 (5.7%)
Residential status	
Live alone	7 (5.0%)
Live with family	133 (95.0%)
Employment status	
Currently employed	16 (11.2%)
Unemployed	8 (5.7%)
Retired	108 (77.1%)
Unknown	8 (5.7%)
Smoking habit	
Never smoked	113 (80.7%)
Smoking	8 (5.7%)
Former smoker	19 (13.6%)
Self-reported medical history	
Coronary heart disease	46 (32.9%)
Diabetic mellitus	25 (17.9%)
Hypertension	52 (37.1%)
Hyperlipidaemia	17 (12.1%)
Exercise experience	
Weekly exercise frequency (days)	5.4 ± 2.6
Average duration of exercise (min/day)	66.6 ± 40.1
Weekly total exercise time (h/week)	7 ± 4.9
Previous Tai Chi experience	51 (36.4%)
TCSE total score (possible range: 0–100)	62.1 ± 31.1
TCSE barriers (possible rage: 0–100)	48.7 ± 40.0
TCSE performance (possible rage: 0–100)	86.3 ± 25.9

Note: BMI: body mass index; CHD: coronary heart disease; SD: standard deviation; TCSE: Tai Chi exercise self-efficacy.

**Table 3:** Item analysis for the TCSE scale (Mandarin Chinese version).

Item Stems	Corrected Item-Total Correlation	Cronbach’s Alpha if Item Deleted
**TCSE Barriers: I believe I can perform Tai Chi exercise three times per week, even if……**		
The weather was bothering me?	0.902	0.966
I am bored by Tai Chi?	0.927	0.965
I feel pain or discomfort when doing Tai Chi?	0.917	0.965
I have to perform Tai Chi alone?	0.900	0.966
I do not enjoy Tai Chi?	0.925	0.965
I am too busy with other activities?	0.912	0.965
I feel tried?	0.921	0.965
I feel stressed?	0.935	0.965
I feel depressed?	0.932	0.965
**TCSE Performance: I believe I can move my whole body in a slow, rhythmical fashion continuously….**		
For 2 min	0.565	0.972
For 5 min	0.581	0.971
For 10 min	0.617	0.971
For 20 min	0.679	0.970
For 30 min	0.713	0.970

Note: TCSE: Tai Chi exercise self-efficacy.

**Table 4:** Known-group comparisons between participants with prior Tai Chi experience and those without prior Tai Chi experience (*n* = 140).

Item	PPTCE (*n* = 51, Mean ± SD)	POTCE (*n* = 89, Mean ± SD)	*p*-Value
TCSE barriers	83.01 ± 24.94	29.02 ± 33.24	<0.001 ^†^
TCSE performance ^‡^	100.00 ± (96.00, 100.00)	92.00 ± (79.00, 100.00)	<0.001 ^§^
TCSE total score	87.23 ± 20.54	47.72 ± 26.77	<0.001 ^†^

Note: PPTCE: participants with prior Tai Chi experience; POPTCE: participants without prior Tai Chi experience; TCSE: Tai Chi exercise self-efficacy. ^†^: independent T-test; ^‡^: TCSE performance scores presented as median ± (lower quartile, upper quartile); ^§^: Mann–Whitney U Test.

## Data Availability

The data presented in this study are available on request from the corresponding author. The data are not publicly available for privacy reasons.
